# Compliance Investigation of Honey‐Packaged Food Labels and Claims in Saudi Arabia

**DOI:** 10.1155/ijfo/7113620

**Published:** 2025-11-11

**Authors:** Lulu A. Almutairi, Amani S. Alqahtani

**Affiliations:** ^1^ Saudi Food and Drug Authority, Riyadh, Saudi Arabia, sfda.gov.sa

**Keywords:** compliance, honey claims, honey labeling, regulation, Saudi Arabia

## Abstract

**Background:**

Honey provides various nutritional, health, and economic advantages, making it crucial to oversee its production and import. Thus, the Saudi Food and Drug Authority (SFDA) regulates and monitors honey products. This study assesses the compliance of honey products with the SFDA′s food labeling and claims regulations.

**Methods:**

This observational cross‐sectional study was aimed at assessing the compliance of the honey products labeling requirements set by SFDA. The data of this study were collected by surveying the package labeling information of selected prepackaged honey. The labeling compliance was assessed by using a comprehensive checklist that takes into account the various aspects of honey products labeling requirements. The claims′ compliance was assessed based on SFDA health and nutritional claims technical regulation.

**Results:**

The present study involved 306 locally distributed honey products. Natural honey and Sidr honey were the most common types included in the sample. Out of the 10 compliance components, only four had a compliance rate of 90% or above. Brand name was the most commonly complied component on all products′ labels at 100%, followed by food additives at 99.67%. Only 1.96% of the honey samples carried health or nutritional claims; nutritional claims were found to be 100% compliant, and 50% of the written health claims were identified as prohibited claims.

**Conclusion:**

The assessment revealed both strengths and areas for improvement. While there was high compliance with nutritional claims and accurate product naming, there were notable issues with health claims and batch numbering. These findings underscore the importance of ongoing monitoring and regulatory oversight.

**Summary**

• Brand name was the most consistently compliant component across all products′ labels.
• Warning statements and batch numbering were among the least compliant components.
• Only a small percentage of honey products contained health or nutritional claims.

## 1. Introduction

Honey is considered a natural product, made by bees from the nectar of flowers, from the secretions of living plant parts or the exudates of sap‐sucking insects of the order (Hemiptera) and stored by bees in the hexagonal cells of honeycombs [1]. Honey has been known since ancient times for its wide uses in the food and pharmaceutical fields, contributing to many nutritional, health, and economic benefits [1, 2]. Consequently, the Saudi Food and Drug Authority (SFDA) is keen to spread the awareness message among beekeepers, producers, and marketers of honey to follow and support all sound practices in honey marketing and trading [3].

Honey has been identified as one of the most adulterated ingredients throughout history, and it has been frequently falsified and inaccurately represented in terms of floral sources and geographical origin [4]. Therefore, labels have been developed to recognize honey quality linked to origin or production method in order to inform consumers about these specific determinants as well as prevent misleading claims and ensure all manufacturers compete on a level playing field by requiring accurate and consistent information disclosure on food labels [5]. Ensuring the safety and accurate labeling of food is crucial for consumer well‐being, taking into account that package labels serve as the primary point of contact between consumers and food business operators [6].

Regulating honey products can be challenging for authorities due to various reasons. For example, it is becoming increasingly popular to buy honey directly from beekeepers, which increases the variety of sources from which consumers can purchase honey products [7]. Another big problem is that honey sold in supermarkets is frequently falsified, even though falsifying or other forms of tampering with honey are strictly prohibited [8]. According to a study conducted on 320 samples into the fraudulent practices of honey, 46% of honey imported into the EU is suspected of not complying with the provisions of the “honey directive”, which introduces several key provisions aimed at improving transparency, quality, and consumer trust in honey products labeling and composition [9]. Moreover, measuring the compliance of honey labeling and safety requirements presents another challenge for authorities [5]. With the continuous evolution of ingredients and processing techniques, regulatory frameworks must be regularly updated, placing growing demands on resources needed to ensure compliance and effectively oversee the honey market. The resources include trained personnel, laboratory testing, and data analysis capabilities [5].

In Saudi Arabia, honey products are regulated under the honeybee breeding system issued by Royal Decree No. M/15, whereas the SFDA is the national body responsible for ensuring the safety and quality of honey products and establishing the related regulations and standards [10]. The SFDA is taking steps to regulate and ensure compliance with all food products, including honey. The honey standards covered by the SFDA include many aspects related to honey products labeling, processing methods, production facilities, and honey content, such as additives and taste or flavor. The standards aim to ensure the quality and authenticity of available honey [3]. In terms of honey products labeling, the SFDA has a set of requirements for honey products along with the general food labeling requirements. Some of the requirements include that a product must be labeled as “honey,” and if the source was based on a geographical area, the country of origin must be stated clearly. In addition, the honey product shall not have or use any food additives [1].

This study is aimed at describing honey products′ labeling content and assessing the compliance of the labeling and claims based on the SFDA technical regulations. To our knowledge, there are no existing studies that evaluated the honey labeling and claims in Saudi Arabia. Given that honey is often subject to mislabeling or misleading health claims, this research advances scientific understanding by providing an empirical basis for evaluating the authenticity and labeling accuracy of honey products in Saudi Arabia. Additionally, it may highlight gaps or inconsistencies in compliance, providing data that can guide policy adjustments and improve consumer protection. This research also offers valuable insights at a global level as honey is widely traded internationally, and standards for labeling and claims vary significantly across countries. The findings could serve as a benchmark for other countries, especially those in the region, and contribute to harmonizing labeling standards and practices globally.

## 2. Materials and Methods

### 2.1. Study Design

This study employed an observational cross‐sectional design to assess the compliance of the honey labeling requirements set by the SFDA by surveying the package labeling information of selected prepackaged honey products available in the Saudi market.

### 2.2. Data Source, Entry, and Validation

Primary data for this study, in the form of photos, were extracted from the SFDA food registration database for products registered in 2021. The overall study sample consists of 306 varieties of prepackaged honey products from different brands, regions, and flower sources. All extracted photos included all sides of the food package to ensure all necessary information was captured. This information covers multiple variable sections, including all mandatory and conditionally mandatory or optional details required on food labels by the SFDA [11]. These include the product name and type, front and back labels, ingredient lists, nutrition facts table, country of origin, and any health claims made on the product package label. To avoid duplication, only unique honey products were included in the study, meaning only one product of the same brand, type, and size was selected for analysis. Then, the labeling information from the photos was manually entered into a Microsoft Excel spreadsheet by trained human nutrition postgraduate researchers. For data validation, after every 100 product entries, a different researcher reentered 10% of the data for verification. They then compared the two entries to address any differences or assess whether there were any incorrect values.

### 2.3. Data Variables

**Figure 1:**
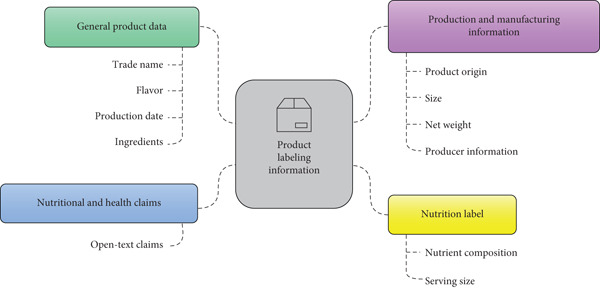
Products′ labeling categorization.

The variables gathered from product package labels were categorized into four sections. The first section concerns general product data, such as the product′s trade name, flavor, production date, and ingredients. The second section relates to production and manufacturing information, including the product′s origin, size, net weight, and producer information. The third section is about the nutrition label, which consists of the nutrient composition and serving size from the nutrition fact panel. Finally, the fourth section is about nutritional and health claims. It consists of open‐text variables that include the claims appearing on the product. Any claims that fall within the definitions of the Saudi Technical Regulation No. SFDA.FD 2333:2020 “Requirements for Health and Nutrition Claims” are recorded as stated on the product. Figure 1 visualizes the sections and categories of the product′s information.

### 2.4. Assessment Criteria

Our approach involves the use of a comprehensive checklist that takes into account the various aspects of honey products labeling. The checklist criteria are determined based on two critical technical regulations that outline the mandatory information, conditionally mandatory information, and optional information required to be included on honey packaging. Our evaluation process focuses solely on the mandatory labeling and conditionally mandatory elements of each product label. This approach ensures that we assess the compliance of each product label accurately and comprehensively. For the honey package labeling assessment, we used the Honey Technical Regulation “SFDA.FD 147:2021” for the checklist (Table 1). For the health and nutrition claims classifications, we used the “SFDA.FD 2333:2020” technical regulation to define the claim types as well as to define the permitted and prohibited health and nutrition claims (classification components are described in Table 2).

**Table 1:** Honey products labeling compliance checklist with description.

**Number of requirements**	**Criteria based on the guidelines**	**Description**
1	Product name	The label must clearly identify the product as “honey” or include the word “honey” in conjunction with the type or floral source if applicable (e.g., “clover honey”). If the source was based on a geographical area, the country of origin must be stated clearly.
2	List of ingredients	If the honey is pure, no ingredient list is required. However, if any other ingredients are added, they must be listed in descending order.
3	Net content	The net weight or volume of the honey must be displayed on the label in metric units (grams or kilograms for weight and milliliters or liters for volume).
4	Producer information	The name and address of the manufacturer or packer shall be declared in the case of the packer is not the manufacture. The name of distributor, importer, exporter, or vendor may be written.
5	Country of origin	The label must specify the country of origin of the honey and it is specifically mandatory if the origin was written with the name.
6	Storage conditions	Any specific storage conditions or instructions must be provided on the label to ensure the quality of the honey.
7	Batch number	The batch or lot number of the honey must be included on the label.
8	Nutritional information	The label should provide nutritional information of the amount of energy, protein, fat, saturated fat, trans fat, cholesterol, sodium, total of sugars, and added sugar.
9	Warning statements	The label must include warning statements for foods or ingredients that may cause health risks for some consumers, such as a warning for infants under 1 year of age not to consume honey.
10	Food additives	The honey must not contain any natural or artificial food additives or substances and does not contain any additives other than honey.

**Table 2:** Claims assessment criteria and description.

**Types of claim**	**Definition**	**List of permitted claims**	**Example of prohibited claims**
Nutritional claims	Any claim that states, suggests, or implies that a food has particular beneficial nutritional properties	• Essential fatty acids are needed for normal growth and development of children • Source of [name of vitamin/s] and/or [name of mineral/s] • Contains [name of the nutrient or other substance]	• Claims which refer to recommendations of individual doctors or health professionals • Claims which suggest that health could be affected by not consuming the food • Claims show that a food can be used in the prevention, alleviation, treatment, or cure of a disease
Health claims	Any claim that states, suggests or implies that a relationship exists between a food category, a food or one of its constituents and health.		

### 2.5. Analysis Plan

We performed a descriptive analysis to summarize the study′s variables. For categorical variables, we calculated frequencies and percentages. For continuous variables, such as nutritional values per 100 g, we determined the central tendency (mean and median) and dispersion (standard deviation, minimum, and maximum). To ensure the robustness of the descriptive analysis, outliers in the nutritional data were identified using the interquartile range (IQR) method. The few identified values were then replaced with the median of their respective distributions to prevent skewing the summary statistics. Additionally, we evaluated each component of the assessment′s checklist as a yes/no response to determine whether the components were present or absent. The mean compliance with labeling requirements was calculated by determining the presence of 10 requirements for each product. We also reviewed the claims recorded in the dataset to determine their compliance and presented the results as numbers and percentages. All statistical analyses were performed via IBM SPSS V.23.0 software and Excel.

## 3. Results

### 3.1. Descriptive Analysis of the Sample

The present study involved the analysis of 306 locally distributed honey products intended for human consumption. Among the products analyzed, natural honey and Sidr honey were the most common types included in the sample, accounting for 38% of the total sample. More information about the honey types can be found in Table 3. Approximately 199 honey products (65%) were imported, while 11 samples (3.6%) did not disclose their country of origin (Table 4). A detailed breakdown of the countries of origin for the honey samples is provided in Table S1 (available here). More details of the country names of the honey samples can be found in Table S1. Fifteen different nutrient values were reported across various honey products. Calories (280, 91.50%), carbohydrates (267, 87.25%), total fat (257, 84%), protein (257, 84%), and sodium (206, 67.32%) were the most frequently listed nutrients. The average calories per 100 g of honey are 303 calories, with a range of 187–400 calories. Additionally, the mean sugar content per 100 g of honey is 75.68 g, with a range of 23–83 g. More of the nutritional information of honey is presented in Table 5.

**Table 3:** Honey type as labeled by the producer.

**Honey name**	**N** **(%)**
Natural honey	61 (19.93)
Sidr honey	56 (18.30)
Multiflora honey	34 (11.11)
Acacia honey	16 (5.22)
Manuka honey	25 (8.16)
Black forest honey	15 (4.90)
Mixed and flavored	13 (4.24)
Sumar honey	13 (4.24)
Talh honey	12 (3.92)
Black seed honey	9 (2.94)
Thyme honey	6 (1.96)
Others^a^	46 (15)
Total	306

^a^Honey types of less than three products are grouped as “others”; some of the honey types included in this group are “white honey, thistles honey, and *Moringa* honey.”

**Table 4:** Characteristics of honey products origin.

**Origin**	**N** **(%)**
Domestic	96 (31.40)
Imported	199 (65)
Not available	11 (3.60)
Total	306

**Table 5:** Descriptive analysis of the nutrients available in honey products based on the values declared on the label (*N* = 306).

**Nutrients**	**Number of products**	**Mean/100 g**	**SD**	**Median/100 g**	**Min–max/100 g**
Calories	280	303	27	300	187–400
Carbohydrates	267	77.73	8.59	80	13–86.5
Total fat	257	0.06	0.02	0	0–1
Protein	257	0.4	0.83	0.05	0–4.9
Sodium	206	0.01	0.07	0	0–0.9
Sugar	165	75.68	6.83	78	23–83
Saturated fat	153	0.1	0.95	0	0–10.48
Fiber	119	0.06	0.34	0	0–2.49
Trans fat	117	0.03	0.1	0	0–0.50
Cholesterol	117	0	0	0	0–0
Added sugar	74	0.01	0.043	0	0–0.50
Calcium	32	1.6	0.92	0	0–4.5
Iron	30	0.5	0.26	0	0–1.3
Potassium	30	0	0	0	0–0.2
Vitamin C	14	2	0.5	2.4	0–2.4

### 3.2. Honey Products′ Food Labeling Compliance

Out of 10 compliance components, only four had a compliance rate of 90% or higher. Brand name (306, 100%) was the most consistently compliant component on all products′ labels, followed by food additives (305, 99.67%). The majority of the products (299, 97.71%) were found to comply with adding the net content of the honey product. In addition, some essential components, such as storage conditions (228, 47.50%), list of ingredients (212, 69.28%), and batch number (146, 47.71%), were notably missing. Out of all the analyzed products, only 105 (34.31%) had warning statements. More details are shown in Table 6.

**Table 6:** Classification of honey products compliance based on SFDA guidelines for labeling (*N* = 306).

**Labeling requirements**	**Compliant (%)**	**Noncompliant (%)**
Product name	306 (100)	0
Food additives	305 (99.67)	1 (0.32)
Net content	299 (97.71)	7 (2.28)
Country of origin	295 (96.40)	11 (3.60)
Nutritional information	281 (91.83)	25 (8.17)
Producer information	255 (83.33)	51 (16.66)
Storage conditions	228 (74.50)	78 (25.50)
List of ingredients	212 (69.28)	94 (30.72)
Batch number	146 (47.71)	160 (52.29)
Warning statements	105 (34.31)	201 (65.69)

### 3.3. Honey Products′ Health and Nutrition Claims Compliance

Of the 306 analyzed products, only 1.96% of them carried health or nutritional claims. Nutritional claims constituted 33.33%, while health claims accounted for 66.66%. In terms of compliance assessment, nutritional claims were found to be 100% compliant with the list of allowed claims. However, 50% of the written health claims were identified as prohibited. Table 7 provides examples of the claims found on the products.

**Table 7:** Compliance analysis of the available claims with regard to the allowed and prohibited claims as per the guidelines provided by SFDA.

**Types of claim**	**N**	**Prohibited claim**	**Allowed claim**	**Example**
Nutritional claims	2	0	2	It contains, on average, 18 times the antioxidants of regular honey.
Health claims	4	2	2	This food does not contain much saturated fat, cholesterol, dietary fiber, added sugar, or protein. For children, half a tablespoon should be given three times a day, 1 h before meals. It can be taken with milk, twice a day—once in the morning and once in the evening.
Total	6	2	4	—

## 4. Discussion

The compliance level with honey products labeling guidelines varied significantly across different components based on the study assessment. Overall, only four components had a compliance rate of 90% or higher. Compared to other studies, the specific components with which products complied varied; however, overall compliance rates consistently remained low. A study conducted in Fiji found only 22% of the products fully complied with the nutritional labeling requirements in the honey group [12]. Moreover, another study found that 89.32% of honey packages lacked at least one of the mandatory pieces of information required in the regulations, and only 10.7% of honey labels met the legal labeling criteria [13].

The product name component exhibited the highest level of compliance, indicating that manufacturers are generally accurate in naming their honey products. This is similar to other findings where the product name is the most commonly present item on the label [13]. This could be attributed to manufacturers′ practices, where the product′s name is prominently featured, and the honey type is important to consumers. Therefore, it is crucial for manufacturers to have the name clearly stated on the label regardless of the regulation requirements. Additionally, our study revealed high compliance regarding food additives in honey products labeling, suggesting that manufacturers are generally adhering to regulations in this aspect. However, it is important to note that this compliance is based on the information provided on the product labels, particularly the ingredients list, rather than direct quality lab tests. While this indicates promising regulatory adherence, it also highlights the need for ongoing monitoring and analysis of honey products. This is crucial due to the potential risks associated with consuming honey with undisclosed or excessive food additives. Honey has been identified as one of the most adulterated ingredients throughout history, and it has often been substituted or misrepresented [4, 14, 15]. Honey is often adulterated for different reasons, such as to enhance the taste by adding sugars according to the consumer′s preference. Alternatively, it could be done to increase financial profits by mixing low‐quality honey with expensive honey [14].

Compliance with these four components may reflect positively on regulatory efforts and industry practices. However, this was contrasted by the warning statement and batch number components, which emerged as the least compliant aspects. This finding raises concerns about the need for greater emphasis on informing consumers about critical aspects of honey, such as the presence of allergens or the necessity of caution for infants. It is worth noting that over half of the mothers (52%) in a study conducted in Saudi Arabia were found to have given honey to their infants before they turned 12 months old, with a majority providing honey between the ages of 4 and 7 months. This highlights the significance of having a warning statement to safeguard infants [16]. Furthermore, complying with batch number requirements is important for ensuring product quality, consumer safety, and regulatory compliance in the honey industry [17]. This is particularly important in the event of a product recall, as the batch numbers are essential for tracking and monitoring the quality of honey products. Without batch numbers, it becomes challenging to trace products back to specific production runs in case of quality issues or contamination [18].

The analysis of health and nutritional claims in honey products labeling revealed noteworthy insights. Only a small percentage of the analyzed products featured such claims. Nutritional claims were found to be fully compliant indicating that manufacturers are adhering to the permitted claims. Nevertheless, it is important to ensure that claims are based on accepted scientific evidence and meet registration requirements. In terms of health claims, the study revealed a concerning finding where half of the written health claims were classified as prohibited claims, suggesting a need for stricter adherence to regulatory guidelines. Similarly, a study conducted in Bosnia and Herzegovina found that 7.6% of the honey products included in the study contained health claims such as “solve urological problems” and “treat coughs and respiratory problems” [19]. Another study analyzed the type of claims observed in various food products and found that most of the used noncompliant or unauthorized wording [20].

Lastly, the descriptive analysis of nutritional information on honey labels reveals significant inconsistencies in the availability and presentation of data. While calorie and carbohydrate values are almost reported on all products, the declaration of other key nutrients such as protein, fiber, and various micronutrients is highly variable and often absent. The frequent reporting of a zero median for many nutrients, including fat, protein, and sodium, coupled with a wide range between minimum and maximum values, suggests that while some products may contain these elements, their presence is not a consistent feature across the sampled honey products. This variability highlights a lack of standardized labeling practices, which can impede consumers from understanding the true nutritional profile of honey beyond its primary components of carbohydrates and sugars.

It is recommended that food authorities develop specific guidelines for honey products, considering their cultural significance, health benefits, and ongoing regulatory concerns around claims [2]. These guidelines should aim to enhance consumer understanding about honey quality and enable them to make informed decisions when purchasing it. Taking into account the large price variation between honey products in Saudi Arabia [21], it is essential to provide consumers with the necessary information to help them evaluate authenticity and quality more effectively. For example, in Canada, honey is graded based on several factors, including its color, flavor, aroma, and moisture content. The Canadian grading system for consumer prepackaged honey includes four primary color classes: white, golden, amber, and dark. Canada No. 3 is the highest quality grade, with specific requirements for each of these color classes (Canadian Food Inspection [22]). Additionally, the United States Department of Agriculture (USDA) has a specific honey grading system that considers several factors, such as moisture content, clarity, flavor, and absence of defects. The grading is determined through sensory evaluation and laboratory analysis, and the grade is indicated on the honey label [23].

Limitations of the study include that the compliance evaluation was based on the visible information in the images and based on the status of the product when applied for approval in the SFDA, which might affect the generalizability of the study. Moreover, our assessment was based on the declared information rather than laboratory tests, which assumes that the information provided there remains the possibility that the information may be misleading or inaccurate. Regarding study strengths, the honey sample included in this study was high compared to other studies that analyzed honey or food labels in general.

## 5. Conclusion

The assessment of honey products′ labeling compliance revealed both strengths and areas for improvement. Although there was high compliance with nutritional claims and accurate product naming, notable shortcomings were identified in the areas of health claims, warning statements, and batch numbering. Furthermore, the study found significant inconsistencies in the availability and presentation of nutritional values on honey product labels. These findings underscore the importance of ongoing monitoring and regulatory oversight to ensure that honey products′ labeling remains transparent, accurate, and conducive to informed consumer choices.

## Disclosure

All authors reviewed the results and approved the final version of the manuscript. The authors report that the conclusions reached in this article are based on the personal scientific interpretations of the authors and do not necessarily represent the opinion of SFDA.

## Conflicts of Interest

The authors declare no conflicts of interest.

## Author Contributions

Lulu A. Almutairi: designing the study and data analysis and drafting the manuscript. Dr. Amani S. Alqahtani: designing the study and supervising all study phases.

## Funding

No funding was received for this manuscript.

## Supporting information


**Supporting Information** Additional supporting information can be found online in the Supporting Information section. It contains a frequency table detailing the country of origin for each honey sample included in the study.

## Data Availability

Data are available upon request from the corresponding author.

## References

[1] Saudi Food and Drug Authority , SFDA.FD 147:2021 (Honey), 2021, https://www.gso.org.sa/store/standards/GSO:811154/GSO147:2022.

[2] Samarghandian S. , Farkhondeh T. , and Samini F. , Honey and Health: A Review of Recent Clinical Research, Pharmacognosy Research. (2017) 9, no. 2, 121–127, 10.4103/0974-8490.204647, 2-s2.0-85018668516. 28539734 PMC5424551

[3] Saudi Food and Drug Authority , Safeguarding Saudi Honey: Exploring the Cultural, Regulatory and International Dimensions of Honey Production in Saudi Arabia, 2024, https://sfda.gov.sa/sites/default/files/2024-03/SFDA_Honey.pdf.

[4] Ahmad N. N. and Khairatun S. N. , Exploring Fraudulent Honey Cases from Readily Available Food Fraud Databases, GATR Global Journal of Business Social Sciences Review. (2021) 9, no. 2, 99–113, 10.35609/gjbssr.2021.9.2(1).

[5] Mădaş M. N. , Mărghitaş L. A. , Dezmirean D. S. , Bobiş O. , Abbas O. , Danthine S. , Francis F. , Haubruge E. , and Nguyen B. K. , Labeling Regulations and Quality Control of Honey Origin: A Review, Food Reviews International. (2020) 36, no. 3, 215–240, 10.1080/87559129.2019.1636063.

[6] FAO , Handbook on Food Labelling to Protect Consumers, 2016, In Food and Agriculture Organization of the United Nations. https://www.fao.org/3/i6575e/i6575e.pdf.

[7] Zavodna L. S. and Pospisil J. Z. , Honey Bee: A Consumer’s Point of View, Environmental & Socio-Economic Studies. (2016) 4, no. 3, 26–32, 10.1515/ENVIRON-2016-0015.

[8] Stukker K. , Honey, What Does Your Label Tell? A Study on Perceived Authenticity in the Honey Industry, and the Influence of Labelling on the Purchase Intention [Wageningen Universty], 2021, https://edepot.wur.nl/586058.

[9] European Parliament and Council , Directive (EU) 2024/1438 of the European Parliament and of the Council of 14 May 2024 Amending Council Directives 2001/110/EC Relating to Honey, 2001/112/EC Relating to Fruit Juices and Certain Similar Products Intended for Human Consumption, 2001/113/EC Relating to Fruit Jams, Jellies and Marmalades and Sweetened Chestnut Purée Intended for Human Consumption, and 2001/114/EC Relating to Certain Partly or Wholly Dehydrated Preserved Milk for Human Consumption, 2024, Official Journal of the European Union. https://eur-lex.europa.eu/legal-content/EN/TXT/?uri=OJ:L_202401438.

[10] Saudi Food and Drug Authority , Honey Guideline, 2021.

[11] GCC Standardization Organization , Requirements of Nutritional Labeling - GSO 2233:2021. Standards Store, 2021, https://www.gso.org.sa/store/standards/GSO:781113/GSO2233:2021?lang=en.

[12] Shahid M. , Waqa G. , Pillay A. , Kama A. , Tukana I. N. , McKenzie B. L. , Webster J. , and Johnson C. , Packaged Food Supply in Fiji: Nutrient Levels, Compliance With Sodium Targets and Adherence to Labelling Regulations, Public Health Nutrition. (2021) 24, no. 13, 4358–4368, 10.1017/S136898002100224X. 34008486 PMC10195241

[13] Vidakovic Knezevic S. , Vranesevic J. , Pelic M. , Knezevic S. , Jaksic S. , Zivkov-Balos M. , and Ljubojević Pelic D. , Current Information Levels on Honey Labels in Vojvodina, IOP Conference Series: Earth and Environmental Science. (2019) 333, no. 1, 012112, 10.1088/1755-1315/333/1/012112.

[14] Fakhlaei R. , Selamat J. , Khatib A. , Razi A. F. A. , Sukor R. , Ahmad S. , and Babadi A. A. , The Toxic Impact of Honey Adulteration: A Review, Foods. (2020) 9, no. 11, 1538, 10.3390/FOODS9111538. 33114468 PMC7692231

[15] Jaafar M. B. , Othman M. B. , Yaacob M. , Talip B. A. , Ilyas M. A. , Ngajikin N. H. , and Fauzi N. A. M. , A Review on Honey Adulteration and the Available Detection Approaches, International Journal of Integrated Engineering. (2020) 12, no. 2, 125–131, 10.30880/ijie.00.00.0000.00.0000, 2-s2.0-85065486021.

[16] Bamumin A. , Bamumin S. , Ahmadini H. A. , Alhindi Y. , Alsanosi S. , Alqashqari H. , Esheb G. , Ayoub N. , and Falemban A. , Knowledge, Attitude and Practice Among Mothers on the Relationship Between Honey and Botulism in Saudi Arabian Infants: A Cross-Section Study, Annals of Medicine. (2023) 55, no. 2, 2279746, 10.1080/07853890.2023.2279746. 37943711 PMC10653683

[17] Aung M. M. and Chang Y. S. , Traceability in a Food Supply Chain: Safety and Quality Perspectives, Food Control. (2014) 39, no. 1, 172–184, 10.1016/J.FOODCONT.2013.11.007, 2-s2.0-84896964269.

[18] Dupuy C. , Botta-Genoulaz V. , and Guinet A. , Batch Dispersion Model to Optimise Traceability in Food Industry, Journal of Food Engineering. (2005) 70, no. 3, 333–339, 10.1016/J.JFOODENG.2004.05.074, 2-s2.0-17944379627.

[19] Alibabić V. , Mujić I. , Rudić D. , Bajramović M. , Jokić S. , Šertović E. , and Ruţnić A. , Labeling of Food Products on the B&H Market and Consumer Behavior Towards Nutrition and Health Information of the Product, Procedia-Social and Behavioral Sciences. (2012) 46, 973–979, 10.1016/j.sbspro.2012.05.233.

[20] Coates E. , Pentieva K. , and Verhagen H. , The Prevalence and Compliance of Health Claims Used in the Labelling and Information for Prepacked Foods Within Great Britain, Food. (2024) 13, no. 4, 10.3390/foods13040539. PMC10887619 38397515

[21] Ismaiel S. , Kahtani S.Al, Adgaba N. , Al-Ghamdi A. A. , and Zulail A. , Factors That Affect Consumption Patterns and Market Demands for Honey in the Kingdom of Saudi Arabia, Food and Nutrition Sciences. (2014) 2014, no. 17, 1725–1737, 10.4236/FNS.2014.517186.

[22] Agency C. F. I. , Canadian Grade Compendium: Volume 6 - Honey, 2021, Goverment of Canada. https://inspection.canada.ca/about-the-cfia/acts-and-regulations/list-of-acts-and-regulations/documents-incorporated-by-reference/canadian-grade-compendium-volume-6/eng/1523388139064/1523388171017.

[23] Agricultural Marketing Service , Extracted Honey Grades and Standards, 2024, USDA. https://www.ams.usda.gov/grades-standards/extracted-honey-grades-and-standards.

