# Supplementary material for: Compliance Investigation of Honey‐Packaged Food Labels and Claims in Saudi Arabia
Source: Int J Food Sci. 2025 Nov 11;2025:7113620. doi: 10.1155/ijfo/7113620 (PMC12611324; doi:10.1155/ijfo/7113620)
Supplement: Supplementary file 1 — Supporting Information Additional supporting information can be found online in the Supporting Information section. It contains a frequency table detailing the country of origin for each honey sample included in the study. [file IJFO-2025-7113620-s001.docx]

**Table 1.** Frequency and Percentage of Honey Samples by Country of Origin

| ***Country*** | ***N (%)*** |
| --- | --- |
| KSA | 96 (31.37) |
| Kuwait | 28 (9.15) |
| Germany | 22 (7.18) |
| New Zealand | 19 (6.20) |
| Pakistan | 16 (5.22) |
| Spain | 15 (4.90) |
| Egypt | 14 (4.57) |
| India | 12 (3.92) |
| Yemen | 12 (3.92) |
| Greece | 10 (3.26) |
| France | 8 (2.61) |
| Australia | 7 (2.28) |
| UAE | 7 (2.28) |
| UK | 5 (1.63) |
| Italy | 5 (1.63) |
| China | 3 (0.98) |
| Hungary | 3 (0.98) |
| Romania | 3 (0.98) |
| Turkey | 2 (0.65) |
| Argentina | 1 (0.32) |
| Portugal | 1 (0.32) |
| Bulgaria | 1 (0.32) |
| Georgia | 1 (0.32) |
| Russia | 1 (0.32) |
| Syria | 1 (0.32) |
| Kazakhstan | 1 (0.32) |
| Holland | 1 (0.32) |
| ***Total*** | ***306*** |
